# Supplementary material for: PrimeDesign software for rapid and simplified design of prime editing guide RNAs
Source: Nat Commun. 2021 Feb 15;12:1034. doi: 10.1038/s41467-021-21337-7 (PMC7884779; doi:10.1038/s41467-021-21337-7)
Supplement: Supplementary file 1 — Supplementary Information [file 41467_2021_21337_MOESM1_ESM.pdf]

PrimeDesign software for rapid and simplified design of prime editing guide RNAs

Jonathan Y. Hsu, Julian Grünewald, Regan Szalay, Justine Shih, Andrew V. Anzalone, Kin Chung Lam, Max W. Shen, Karl Petri, David R. Liu, J. Keith Joung, Luca Pinello

Supplementary information

- Supplementary Figure 1: Overview of prime editing
- Supplementary Figure 2: Relationship between PBS RNA-DNA melting temperature and prime editing efficiency
- Supplementary Note 1: PrimeDesign input sequence encoding the desired edit
- Supplementary Note 2: PrimeDesign recommended pegRNA and ngRNA designs
- Supplementary Note 3: Design annotations for pegRNAs and ngRNAs
- Supplementary Note 4: Genome-wide and saturating mutagenesis designs
- Supplementary Data 1: PrimeDesign analysis of ClinVar variants
- Supplementary Data 2: Sequences of pegRNAs, nicking sgRNAs, primers, and amplicons used

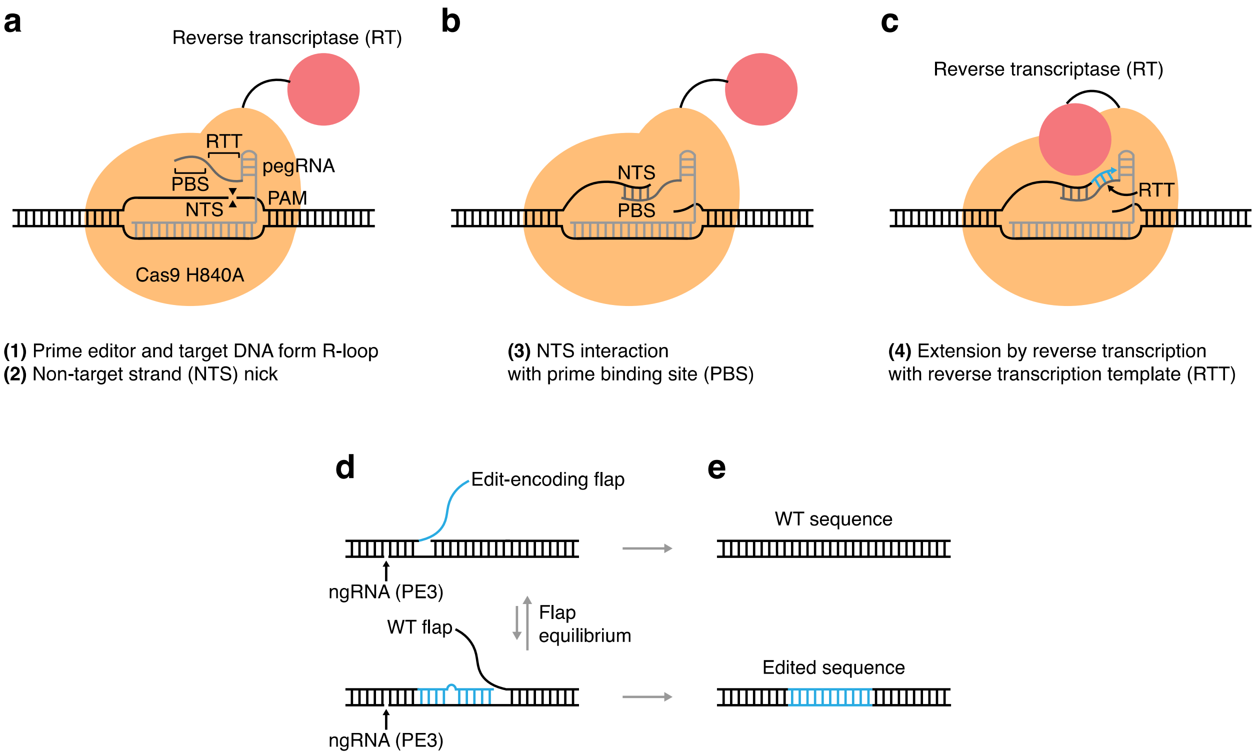

(1) Prime editor and target DNA form R-loop
(2) Non-target strand (NTS) nick

(3) NTS interaction with prime binding site (PBS)

(4) Extension by reverse transcription with reverse transcription template (RTT)

(5) Dissocation of prime editor
(6) Flap competition
(7) Flap excision and ligation
(8) Target strand nick by a ngRNA (PE3)

(9) Preferential repair of the NTS sequence (top strand)

Supplementary Figure 1: Overview of prime editing

a) The prime editor, a fusion protein of a CRISPR-SpCas9 H840A nickase and an engineered Moloney murine leukemia virus reverse transcriptase (MMLV-RT), coupled with a prime editing guide RNA (pegRNA) engages target DNA to form an R-loop and introduces a nick in the non-target strand (NTS). **b)** The 3' end of the NTS interacts with the 3' end of the pegRNA extension, called the primer binding site (PBS), and this stabilized interaction allows for **c)** extension of the NTS by reverse transcription with the reverse transcriptase and reverse transcription template (RTT) of the pegRNA where the edit of interest is encoded in the RTT sequence. **d)** The prime editor system dissociates from its target DNA and the newly-synthesized edit-encoding strand competes with the wild-type (WT) strand for hybridization; flap excision and ligation occurs, and a target strand (TS) nick is introduced with a nicking sgRNA (ngRNA) for the PE3 strategy. **e)**

The NTS sequence is preferentially repaired with the TS nick, resulting in a bias towards successful editing in scenarios where the edit-encoding flap outcompetes the WT flap for genomic incorporation.

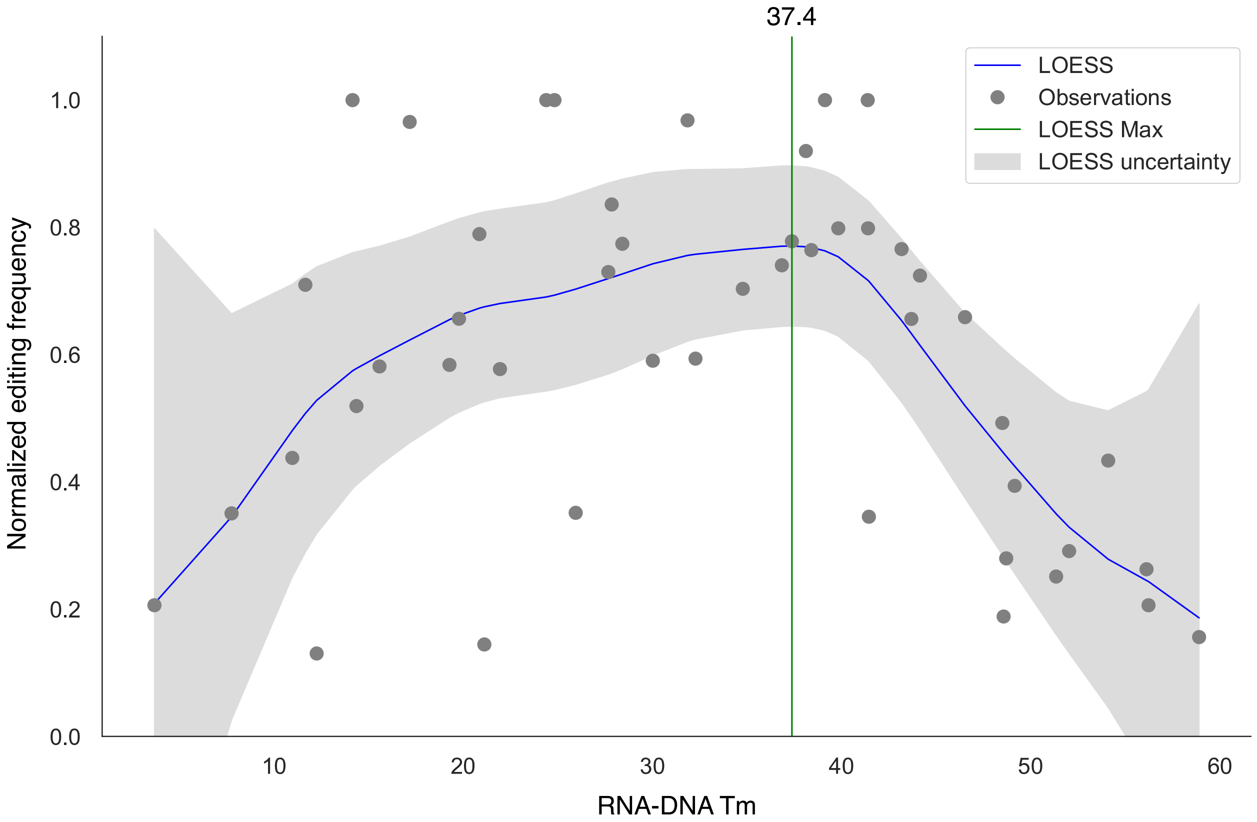

Supplementary Figure 2: Relationship between PBS RNA-DNA melting temperature and prime editing efficiency

Reanalysis of previous data[1] was performed to assess the relationship between the PBS RNA-DNA melting temperature and prime editing efficiency. LOESS was applied and the maximum value of the curve was identified to serve as an initial design recommendation for pegRNA PBS length. The LOESS uncertainty is represented by standard error (SE).

Supplementary Note 1: PrimeDesign input sequence encoding the desired edit

The PrimeDesign input sequence format encodes both the original reference and desired edited sequences. All edits are formatted within a set of parentheses. Substitution edits are encoded by: (*ref*/*edit*), where *ref* is the pre-substitution reference sequence and *edit* is the post-substitution edited sequence. Insertion edits are encoded by: (+*ins*) or (/*ins*), where *ins* is the sequence to be inserted into the reference sequence during the editing event. Deletion edits are encoded by: (-*del*) or (*del*/), where *del* is the sequence to be deleted from the reference sequence during the editing event. Substitution, insertion, and deletion edits can be combined for combination edits. All sequences unaffected by editing remain outside of the parentheses. It is recommended to place the intended edit site near the center of the input sequence and have the total input sequence length be >300 bp to ensure thorough design for prime editing. We provide some examples of input sequences below:

Substitution edit:

CACACCTACACTGCTCGAAGTAAATATGCGAAGCGCGGCCTGGCCGGAGGCGTTCCGCGCCGCCAC
GTGTTCGTTAACTGTTGATTGGTGGCACATAAGCAATCGTAGTCCGTCAAATTCAGCTCTGTTATCCCGG
GCGTTATGTGTCAAATGGCGTAGAACGGGATTGACTGTTTGACGGTAGCTGCTGAGGCGG(G/T)AGAG
ACCCTCCGTCGGGCTATGTCACTAATACTTTCCAAACGCCCCGTACCGATGCTGAACAAGTCGATGCAGG
CTCCCGTCTTTGAAAAGGGGTAAACATACAAGTGGATAGATGATGGGTAGGGGCCTCCAATACATCCAA
CACTCTACGCCCTCTCCAAGAGCTAGAAGGGCACCCTGCAGTTGGAAAGGG

Insertion edit:

CACACCTACACTGCTCGAAGTAAATATGCGAAGCGCGGCCTGGCCGGAGGCGTTCCGCGCCGCCAC
GTGTTCGTTAACTGTTGATTGGTGGCACATAAGCAATCGTAGTCCGTCAAATTCAGCTCTGTTATCCCGG

GCGTTATGTGTCAAATGGCGTAGAACGGGATTGACTGTTTGACGGTAGCTGCTGAGGCGGGA(+GTAA)

GAGACCCTCCGTCGGGCTATGTCACTAATACTTTCCAAACGCCCCGTACCGATGCTGAACAAGTCGATGC

AGGCTCCCGTCTTTGAAAAGGGGTAAACATACAAGTGGATAGATGATGGGTAGGGGCCTCCAATACAT

CCAACACTCTACGCCCTCTCCAAGAGCTAGAAGGGCACCCTGCAGTTGGAAAGGG

Deletion edit:

CACACCTACACTGCTCGAAGTAAATATGCGAAGCGCGCGGCCTGGCCGGAGGCGTTCCGCGCCGCCAC

GTGTTCGTTAACTGTTGATTGGTGGCACATAAGCAATCGTAGTCCGTCAAATTCAGCTCTGTTATCCCGG

GCGTTATGTGTCAAATGGCGTAGAACGGGATTGACTGTTTGACGGTAGCTGCTGAGGCGGGAG(-

AGAC)CCTCCGTCGGGCTATGTCACTAATACTTTCCAAACGCCCCGTACCGATGCTGAACAAGTCGATGC

AGGCTCCCGTCTTTGAAAAGGGGTAAACATACAAGTGGATAGATGATGGGTAGGGGCCTCCAATACAT

CCAACACTCTACGCCCTCTCCAAGAGCTAGAAGGGCACCCTGCAGTTGGAAAGGG

Combination edit:

CACACCTACACTGCTCGAAGTAAATATGCGAAGCGCGCGGCCTGGCCGGAGGCGTTCCGCGCCGCCAC

GTGTTCGTTAACTGTTGATTGGTGGCACATAAGCAATCGTAGTCCGTCAAATTCAGCTCTGTTATCCCGG

GCGTTATGTGTCAAATGGCGTAGAACGGGATTGACTGTTTGACGGTAGCTGCTGAGGCGG(G/T)A(+GT

AA)G(-

AGAC)CCTCCGTCGGGCTATGTCACTAATACTTTCCAAACGCCCCGTACCGATGCTGAACAAGTCGATGC

AGGCTCCCGTCTTTGAAAAGGGGTAAACATACAAGTGGATAGATGATGGGTAGGGGCCTCCAATACAT

CCAACACTCTACGCCCTCTCCAAGAGCTAGAAGGGCACCCTGCAGTTGGAAAGGG

Supplementary Note 2: PrimeDesign recommended pegRNA and ngRNA designs

PrimeDesign provides a pegRNA and ngRNA recommendation to install an edit of interest based on best practices described in Anzalone et al. 2019. The determination of the pegRNA spacer is performed with the following preference: 1) *PAM disrupted* annotation 2) minimization of distance of nick to edit of interest. The determination of the pegRNA PBS length is performed by calculating the RNA-DNA melting temperatures for all possible PBS lengths, and then choosing the PBS length that is closest to our determined value of 37°C (Supplementary Figure 2). The determination of the pegRNA RTT length is performed by calculating the edit length and then constructing an RTT with a certain length of homology downstream of the edit: 1) edit length <= 1 is 10 nt homology downstream 2) 1 < edit length <= 5 is 15 nt homology downstream 3) 5 < edit length <= 10 is 20 nt homology downstream 4) 10 < edit length <= 15 is 25 nt homology downstream and 5) edit length > 15 is 35 nt homology downstream. The determination of the ngRNA spacer is performed with the following preference: 1) *PE3b seed* annotation 2) *PE3b non-seed* annotation 3) *PE3* annotation at a distance as close to 75 bp away from the pegRNA spacer. While the PrimeDesign pegRNA and ngRNA recommendations are informed based on the information to date, we note it should serve as an initial point for design and that further empirical testing of pegRNA and ngRNA designs may be needed to achieve optimal prime editing efficiencies.

Supplementary Note 3: Design annotations for pegRNAs and ngRNAs

PrimeDesign provides important annotations during the design of pegRNAs and ngRNAs. For pegRNAs, the different annotations include: *PAM intact*, *PAM disrupted*, and *PAM disrupted silent mutation*. The *PAM intact* annotation is given to pegRNAs that do not introduce edits into the PAM sequence at positions that have sequence preference, whereas the *PAM disrupted* annotation is given to pegRNAs that introduce sequence modifications at PAM positions that have sequence preference. For coding sequence edits, PrimeDesign offers a functionality to introduce silent mutations to potentially improve editing efficiency and product purity. When this functionality is turned on and the design is available, the *PAM disrupted silent mutation* is provided for suitable pegRNA designs. Importantly, the input sequence must be provided in-frame in order for this function to work properly. We recommend using the amino acid sequence viewer on our PrimeDesign web application to check whether the input sequence is in-frame, and deleting the left-most bases of the input sequence to achieve the correct frame. PrimeDesign uses the GenScript human codon usage frequency table (https://www.genscript.com/tools/codon-frequency-table) and automatically selects the best codon by frequency to introduce the silent mutation.

For ngRNAs, the different annotations include: *PE3*, *PE3b non-seed*, and *PE3b seed*. The *PE3* annotation is given to ngRNAs that have a spacer match to both the original reference and desired edited sequences. The *PE3b non-seed* and *PE3b seed* annotations are given to ngRNAs that have a spacer that only perfectly matches the desired edited sequence, and therefore preferentially nick the non-edited strand *after* edited strand flap resolution. The *PE3b non-seed*

ngRNAs contain sequence mismatches to the original reference sequence outside of PAM-proximal nucleotides 1-10 (seed region), whereas *PE3b seed* ngRNAs contain sequence mismatches to the original reference sequence within the seed region. Spacer mismatches in the seed region severely inhibit target DNA binding and cleavage to a larger degree compared to spacer mismatches outside of the seed region. For this reason, *PE3b seed* ngRNAs may exhibit higher specificity in nicking the non-edited strand *after* edited strand flap resolution and are therefore more suitable for the PE3b strategy compared to *PE3b non-seed* ngRNAs.

Supplementary Note 4: Genome-wide and saturating mutagenesis designs

PrimeDesign offers the ability to perform pooled designs for genome-wide and saturating mutagenesis screen applications. For each edit of interest, PrimeDesign outputs a user-defined number of pegRNAs (unique spacers) and ngRNAs per pegRNA. These designs are ranked according to general guidelines previously established by the Liu group. Hierarchal ranking of pegRNAs is performed by first using the pegRNA annotations (*PAM disrupted* -> *PAM disrupted silent mutation* -> *PAM intact*), and then using pegRNA-to-edit distances (smallest to largest). Hierarchal ranking of ngRNAs is performed by first using the ngRNA annotations (*PE3b seed* -> *PE3b non-seed* -> *PE3*), and then using deviations from a user-defined ngRNA-to-pegRNA distance parameter (default: 75 bp).

PrimeDesign enables streamlined design of saturation mutagenesis studies with prime editing at single-base and single-amino acid resolution. The PrimeDesign input sequence format for saturation mutagenesis applications is the following: (*seq*), where *seq* is the user-defined sequence range of where the saturating mutagenesis will take place. If the "base" option is selected, PrimeDesign will automatically construct all single-base changes (i.e. A -> T,C,G) across the user-defined sequence range. If the "amino acid" option is selected, PrimeDesign will automatically construct all single-amino acid changes (including a stop codon) within the user-defined sequence range. Importantly, the user-defined sequence range must be in-frame in order for this function to work properly. PrimeDesign uses the GenScript human codon usage frequency table (https://www.genscript.com/tools/codon-frequency-table) and automatically selects the best codon by frequency to introduce the amino acid changes.

Supplementary Data 1: PrimeDesign analysis of ClinVar variants

Numbers from the PrimeDesign analysis of ClinVar variants regarding their targetability by prime editing technology.

Supplementary Data 2: Sequences of pegRNAs, nicking sgRNAs, primers, and amplicons used

Sequences of pegRNAs, nicking sgRNAs, and primers used for targeted amplicon sequencing.

References

1. Anzalone, A. V. *et al.* Search-and-replace genome editing without double-strand breaks or donor DNA. *Nature* **576**, 149–157 (2019).
