# Supplementary material for: PrimeDesign software for rapid and simplified design of prime editing guide RNAs
Source: Nat Commun. 2021 Feb 15;12:1034. doi: 10.1038/s41467-021-21337-7 (PMC7884779; doi:10.1038/s41467-021-21337-7)
Supplement: Supplementary file 3 — Reporting Summary [file 41467_2021_21337_MOESM3_ESM.pdf]

nature research

Corresponding author(s): Luca Pinello

Last updated by author(s): Jan 9, 2021

Reporting Summary

Nature Research wishes to improve the reproducibility of the work that we publish. This form provides structure for consistency and transparency in reporting. For further information on Nature Research policies, see our Editorial Policies and the Editorial Policy Checklist.

Statistics

For all statistical analyses, confirm that the following items are present in the figure legend, table legend, main text, or Methods section.

n/a	Confirmed	
☐	☒	The exact sample size (n) for each experimental group/condition, given as a discrete number and unit of measurement
☐	☒	A statement on whether measurements were taken from distinct samples or whether the same sample was measured repeatedly
☒	☐	The statistical test(s) used AND whether they are one- or two-sided<br>*Only common tests should be described solely by name; describe more complex techniques in the Methods section.*
☒	☐	A description of all covariates tested
☒	☐	A description of any assumptions or corrections, such as tests of normality and adjustment for multiple comparisons
☐	☒	A full description of the statistical parameters including central tendency (e.g. means) or other basic estimates (e.g. regression coefficient) AND variation (e.g. standard deviation) or associated estimates of uncertainty (e.g. confidence intervals)
☒	☐	For null hypothesis testing, the test statistic (e.g. F, t, r) with confidence intervals, effect sizes, degrees of freedom and P value noted<br>*Give P values as exact values whenever suitable.*
☒	☐	For Bayesian analysis, information on the choice of priors and Markov chain Monte Carlo settings
☒	☐	For hierarchical and complex designs, identification of the appropriate level for tests and full reporting of outcomes
☒	☐	Estimates of effect sizes (e.g. Cohen's d, Pearson's r), indicating how they were calculated

Our web collection on statistics for biologists contains articles on many of the points above.

Software and code

Policy information about availability of computer code

Data collection	Next-generation sequencing data was collected with Illumina MiSeq (targeted DNA amplicon sequencing).
Data analysis	PrimeDesign was used to design pegRNAs and ngRNAs (https://github.com/pinellolab/PrimeDesign). Amplicon sequencing data were analyzed with CRISPResso version 2.0.42 with HDR mode (https://github.com/pinellolab/CRISPResso2).

For manuscripts utilizing custom algorithms or software that are central to the research but not yet described in published literature, software must be made available to editors and reviewers. We strongly encourage code deposition in a community repository (e.g. GitHub). See the Nature Research guidelines for submitting code & software for further information.

Data

Policy information about availability of data

All manuscripts must include a data availability statement. This statement should provide the following information, where applicable:

- Accession codes, unique identifiers, or web links for publicly available datasets
- A list of figures that have associated raw data
- A description of any restrictions on data availability

All targeted amplicon sequencing data have been deposited under the BioProject accession number PRJNA688137 at the Sequence Read Archive (SRA), accessible at: https://www.ncbi.nlm.nih.gov/bioproject/PRJNA688137. Information related to the clinical variants in the manuscript are available at: https://www.ncbi.nlm.nih.gov/clinvar/.

April 2020

Field-specific reporting

Please select the one below that is the best fit for your research. If you are not sure, read the appropriate sections before making your selection.

☒ Life sciences ☐ Behavioural & social sciences ☐ Ecological, evolutionary & environmental sciences

For a reference copy of the document with all sections, see nature.com/documents/nr-reporting-summary-flat.pdf

Life sciences study design

All studies must disclose on these points even when the disclosure is negative.

Sample size	Sample sizes were determined based on previous literature describing prime editing experiments. Citation: Anzalone, A. V. et al. Search-and-replace genome editing without double-strand breaks or donor DNA. Nature 576, 149–157 (2019).
Data exclusions	No data were excluded.
Replication	Independent replicates (n=3) were performed with different cell passages. All attempts at replication were successful.
Randomization	Mammalian cells used in this study were grown under identical conditions; no randomization was used.
Blinding	Mammalian cells used in this study were grown under identical conditions; blinding was not used.

Reporting for specific materials, systems and methods

We require information from authors about some types of materials, experimental systems and methods used in many studies. Here, indicate whether each material, system or method listed is relevant to your study. If you are not sure if a list item applies to your research, read the appropriate section before selecting a response.

Materials & experimental systems

n/a	Involved in the study	
☒	☐	Antibodies
☐	☒	Eukaryotic cell lines
☒	☐	Palaeontology and archaeology
☒	☐	Animals and other organisms
☒	☐	Human research participants
☒	☐	Clinical data
☒	☐	Dual use research of concern

Methods

n/a	Involved in the study	
☒	☐	ChIP-seq
☒	☐	Flow cytometry
☒	☐	MRI-based neuroimaging

Eukaryotic cell lines

Policy information about cell lines

Cell line source(s)	Source: ATCC; cell lines used: HEK293T (ATCC CRL-3216)
Authentication	STR profiling by ATCC (December 2018)
Mycoplasma contamination	Supernatant was analyzed every 4 weeks using MycoAlert PLUS (Lonza). Cells continuously tested negative.
Commonly misidentified lines (See ICLAC register)	HEK293T are not listed in the ICLAC register (version 10).
