# Supplementary material for: PrimeDesign software for rapid and simplified design of prime editing guide RNAs
Source: Nat Commun. 2021 Feb 15;12:1034. doi: 10.1038/s41467-021-21337-7 (PMC7884779; doi:10.1038/s41467-021-21337-7)
Supplement: Supplementary file 4 — Description of Additional Supplementary Files [file 41467_2021_21337_MOESM4_ESM.pdf]

Title: Supplementary Data 1:
Description: PrimeDesign analysis of ClinVar variants

Title: Supplementary Data 2:
Description: Sequences of pegRNAs, nicking sgRNAs, primers, and amplicons used
